# The hazardous (mis)perception of Self-estimated Alcohol intoxication and Fitness to drivE—an avoidable health risk: the SAFE randomised trial

**DOI:** 10.1186/s12954-021-00567-4

**Published:** 2021-12-07

**Authors:** Jöran Köchling, Berit Geis, Cho-Ming Chao, Jana-K. Dieks, Stefan Wirth, Kai O. Hensel

**Affiliations:** 1 Department of Paediatrics, Faculty of Health, Centre for Clinical and Translational Research (CCTR), Helios University Medical Centre Wuppertal, Witten/Herdecke University, Witten, Germany; 2 Institute of Medical Biometry and Epidemiology (IMBE), Faculty of Health, Witten/Herdecke University, Witten, Germany; 3 University Medical Center Rostock, Department of Paediatrics, University of Rostock, Rostock, Germany; 4 University of Giessen and Marburg Lung Center, German Center of Lung Research (DZL), Justus-Liebig-University Giessen, Giessen, Germany; 5 Department of Paediatric Cardiology and Intensive Care Medicine, University Medical Centre, Georg-August-University, Göttingen, Germany; 6 Department of Paediatrics, Cambridge Biomedical Campus, University of Cambridge, Cambridge, UK

**Keywords:** Perceived alcohol intoxication, Road safety, Public health concern, Alcohol-related road traffic accident, Driving under the influence, DUI, Drink-driving

## Abstract

**Background:**

Worldwide, alcohol-related road traffic accidents represent a major avoidable health risk. The aim of this study was to evaluate the accuracy of self-estimating the degree of acute alcohol intoxication regarding the legal driving limit, and to identify risk factors for misjudgement.

**Methods:**

In this prospective randomised controlled crossover trial, 90 social drinkers (mean age 23.9 ± 3.5 years, 50% female) consumed either beer or wine. Study group subjects were made aware when exceeding the legal driving limit (BrAC = 0.05%). Controls received no information about their BrAC. For crossover, beer or wine were consumed in the opposite order.

**Results:**

39–53% of all participants exceeded the legal driving limit whilst under the impression to be still permitted to drive. Self-estimation was significantly more accurate on study day 2 (*p* = 0.009). Increasing BrAC positively correlated with self-estimation inaccuracy, which was reproducible during crossover. Multiple regression analysis revealed fast drinking and higher alcohol levels as independent risk factors for inaccurate self-estimation.

**Conclusions:**

Social drinkers are commonly unaware of exceeding the legal driving limit when consuming alcohol. Self-estimating alcohol intoxication can be improved through awareness. Dedicated awareness programs, social media campaigns and government advice communications should be utilised to address this avoidable hazard.

*Trial registration* The trial was registered prospectively at the Witten/Herdecke University Ethics Committee (trial registration number 140/2016 on 04/11/2016) and at the DRKS—German Clinical Trials Register (trial registration number DRKS00015285 on 08/22/2018—Retrospectively registered). Trial protocol can be accessed online.

**Supplementary Information:**

The online version contains supplementary material available at 10.1186/s12954-021-00567-4.

## Introduction

Worldwide, alcohol consumption and drunk driving constitute significant avoidable health risks and economic burdens despite decades of health promotion activities [1, 2]. Road traffic injuries have become the leading killer of people aged 5–29 years, and recently, the WHO has pronounced alcohol-related traffic accidents as one of its most important underlying causes [1]. Acute alcohol intoxication causes significant motor function impairments in a dose-dependent manner [3]. This becomes especially hazardous when operating a vehicle under the influence (DUI), as drunk drivers may struggle to keep their vehicle in lane and/or show delayed reaction to external stimuli [4–7]. In a systematic review and meta-analysis of simulated vehicle driving studies, acute alcohol intoxication was shown to significantly impair both lateral and longitudinal vehicle control [4]. Moreover, alcohol intoxication leads to decreased motor coordination, speed of information processing, exaggerated steering responses and information-processing capacity [5, 7]. Importantly, alcohol also has hazardous cognitive impacts that can result in unsafe driving [7]. For instance, alcohol intoxication has been associated with increased risk-taking behaviour in young male participants [8]. Taken together, this leads to a highly increased risk of potentially lethal car accidents and other alcohol-related injuries [9].

Social scenarios that involve alcohol consumption often require drinkers to estimate their level of alcohol intoxication and to determine whether this affects their ability to participate in road traffic. The decision to drive under the influence is strongly associated with the subjective sensation of drunkenness and the self-estimation of one’s blood alcohol concentration (BAC) [10]. This is an alarming realization considering the competence to precisely estimate one’s BAC has been shown to be rather inaccurate [7, 11–13]. In light of the potential dangers involved, the discrepancy between the inability to correctly self-estimate one’s level of alcohol intoxication on the one hand and the widespread and frequent consumption of alcohol on the other hand is rather surprising.

The aim of this study was to assess the participants’ ability to accurately self-estimate their fitness to operate a motor vehicle after drinking alcohol. Further, we tested whether this self-estimation can be learned.

## Methods

### Study design

This study was part of a single-centre parallel randomised controlled matched crossover trial that took place from 1 July 2017 to 26 August 2017. Participant recruitment lasted from September 2016 to June 2017. The RCT consisted of two parts: The (previously reported [14]) first part focused on effects of different alcoholic beverage consumption on next day alcohol-induced hangover symptoms. The here reported second part focused on self-estimation regarding intoxication and fitness to operate a motor vehicle. To increase the power of the study, participants were matched with partners similar in age, sex, BMI and drinking behaviour and then randomised to study and control groups using a balanced allocation (matched design). To eliminate bias and to provide an internal validation of our findings, we used a crossover design with a washout period of ≥ 1 week in between the two interventions. A priori, a statistical power analysis was conducted to determine the study sample size as reported in the first part of the trial [14].

### Study eligibility criteria

Using an online survey, we recruited 272 eligible volunteers. Inclusion criteria were good physical fitness, age 18–60 years, prior consumption of beer and wine, and the availability of matching partners to comply with the matched design. Exclusion criteria were a history of drug or alcohol abuse, aversion to wine or beer, alcohol abstinence or intolerance, Eastern Asian ethnicity—given the common variants of alcohol dehydrogenase (e.g. ADH1B, ADH1C) and acetaldehyde dehydrogenase (e.g. mitochondrial ALDH2 allele) coding genes, signs of hepatopathy (i.e. abnormal liver function blood tests), or history of any of the following: gastritis, bariatric surgery, viral hepatitis, alcoholic liver disease, chronic pain, hepatocellular carcinoma, epilepsy, Korsakov syndrome, Wernicke encephalopathy, thiamine deficiency, immunosuppression, recent infection (i.e. gastrointestinal, respiratory, genitourinary, etc.). Further exclusion criteria were pregnancy or breastfeeding, frequent use of painkillers, the use of medications known to interfere with serum alcohol (i.e. via alcohol dehydrogenase, cytochrome 2E1, aldehyde dehydrogenase, e.g. opioids, antibiotics, nitrates or antidepressants).

### Intervention

The first part of the trial, the study design and methodology specifics have been previously reported in detail [14]. In short, the participants were gathered on two separate intervention days and consumed either beer or wine or both until they reached a maximum breath alcohol concentration (BrAC) of 0.11%. Study group subjects were made aware when having reached the legal driving limit. They drank either beer or wine first and were switched to the other respective beverage after having reached the national legal driving limit of BrAC = 0.05%. On their second study participation day, they drank wine and beer in the opposite order (crossover). Control group subjects were not made aware of reaching/exceeding the legal driving limit. They drank either only beer or wine for the first study intervention and vice versa on study day 2. BrAC was monitored repeatedly using breathalysers. With each measurement, a corresponding self-estimation of the subjective BrAC was inquired and documented.

Before participating in the study, all volunteers were asked to refrain from drinking alcohol for 7 days. On the day of the intervention, the participants were asked to eat and drink water in usual amounts as judged by the volunteers themselves. Before participation, all volunteers provided written informed consent, a detailed medical history and underwent a physical examination, as well as blood and urine sampling. Next, we provided a standardised meal for all participants, as calculated according to age- and sex-specific estimated average energy requirements. All interventions were carried out under medical supervision and in accordance with the declaration of Helsinki. The trial was registered prospectively at the Witten/Herdecke University Ethics Committee (trial registration number 140/2016 on 11/04/2017) and at the German Clinical Trials Register (trial registration number DRKS00015285 on 22/08/2018) in retrospect.

We used a premium lager beer, Pilsner recipe from 1847 by Carlsberg (Hamburg, Germany) with an alcohol content of 5% and served it cold. Carlsberg provided the beer free of charge for this trial but had no role in the design, conduct, or analyses of the study. Furthermore, a 2015 Edelgräfler quality white wine (Chasselas blanc/Johanniter, Zähringer Winery, Baden, Germany; ECOVIN-, Bio-wine- and EU-Bio-certified, DE-ÖKO-039, A.P.-No 2081516) with an alcohol content of 11.1% was used and served cold at the same temperature as the beer.

All participants were explicitly asked to come forward when subjectively having reached the legal driving limit. The first BrAC measurement took place either when study participants reported to have reached this level, or after 45–60 min from the start of alcohol consumption. For safety reasons and to obtain serial measurements, repeated BrAC breathalyser measurements were obtained every 45–60 min throughout the process of alcohol consumption using an AlcoQuant® 6020 + device by EnviteC/Honeywell (Wismar, Germany). To avoid technical artifacts and standardize measurements, an obligatory 15-min nil by mouth interval was adhered to prior to each BrAC measurement—as per manufacturer’s recommendation. Data were included for analysis only when both, a BrAC measurement and a corresponding self-estimation, were documented.

This first analysis focused on the volunteers’ capability to accurately estimate having reached the legal driving limit for alcohol (BrAC = 0.05%). For this purpose, the difference between the measured BrAC and estimated BrAC served as the end point. Further, a comparison between the two intervention days was carried out to evaluate whether a learning effect (improved estimation accuracy) can be observed.

Participants were enquired about their well-being at regular intervals throughout the intervention. Alcohol consumption could be terminated early based on volunteer’s personal preference or in light of safety concerns (i.e. loss of orientation, impaired consciousness, altered balance or gait, feeling unwell, disabling nausea, nystagmus, impaired protection reflexes, dysarthria, prolonged reaction time, other neurologic symptoms, illusionary misjudgement, tachycardia, impairing psychomotor symptoms, respiratory or cardiovascular abnormalities, etc.). All data were collected at Witten/Herdecke University Campus between the 1st of July 2017 and the 26th of August 2017.

### Randomisation

Matched partners separately underwent stratified randomisation according to a predetermined allocation ratio using a six-sided dice operated by JK. Moreover, we further randomised control group subjects by the same means to either only wine or beer for study day 1 (and vice versa for study day 2). The “beverage of the day” was concealed at enrolment and only later disclosed to the volunteers on the intervention day by the respective research assistant under the supervision of KH.

### Biostatistical analyses

To analyse the participants’ self-estimation accuracy, BrAC estimations and corresponding measurements of participants who believed to have reached to legal driving limit (estimated BrAC = 0.05%) were taken into consideration and differences between BrAC measurements and corresponding BrAC estimations were calculated and served as primary end point for this analysis.

$$\Delta = {\text{measurement}} \left[ {{\text{BrAC}}} \right] - {\text{self-estimation}} \left[ {{\text{BrAC}}} \right]$$

Negative values represent an overestimation by the participant (measured BrAC was lower than expected), whilst positive values represent an underestimation by the volunteer (measured BrAC was higher than expected). Objectives were to investigate self-estimation accuracy regarding the legal driving limit, as well as to analyse differences between the two intervention days to evaluate whether a learning effect can be observed.

Differences between measured and estimated BrAC values by study day were described using frequency tables, as well as summary statistics and visualised with box plots. To compare mean differences between both study days, paired *t* tests were used in an explorative way.

We further analysed to which degree self-estimation accuracy changed with rising levels of intoxication. All measurements with corresponding estimates were examined. Differences were calculated as mentioned above and served as secondary end point in this analysis. Data are charted as point clouds with measured BrAC levels and the calculated difference between estimation and BrAC measurements.

To identify risk factors favouring an inaccurate self-estimation, exploratory repeated measurement analyses of covariance (ANCOVA) including fixed and random effects were performed for both study days. Specifically, exploratory repeated measurements ANCOVAs with fixed effects (e.g. beverage order, sex, age, preferred beverage, alcohol consumption rate, etc.) and a random subject effect were carried out for both study days. The dependent variable was the subjects’ estimation of current BrAC (absolute difference of measured and perceived BrAC) with all correct or safe estimations being censored by 0. Type III fixed effect tests were performed to identify risk factors. Estimates for fixed effects were presented including standard error and 95% confidence intervals. The analysis was performed with SAS version 9.4 using PROC GLIMMIX.

## Results

**Fig. 1:**
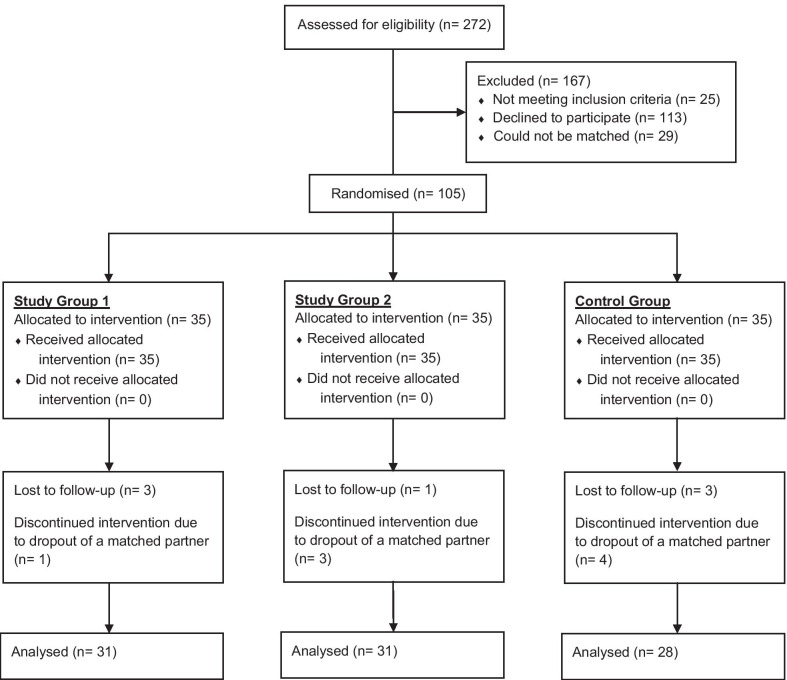
Randomisation and enrolment

The enrolment and randomisation progress are described in detail in our previous report [14] and visualized in Fig. 1. A total of 272 candidates were assessed for eligibility, 247 of those were found eligible, and 105 could be matched and randomised. Ninety completed the trial and were included in the per protocol analyses. As planned, the trial was completed on 26 August 2017, after all data were collected as intended. The difference in planned and final sample size is due to the high loss to follow-up. Main reasons for loss to follow-up by dropped-out candidates were a time overlap of the trial with summer holidays and/or academic examination periods.

Mean age (± standard deviation; interquartile range; min., max.) was 24 (± 3.5; 3; 19, 40) years in the study group and 23.6 (± 3.7; 4; 19, 36) years in the control group. Female sex distribution was 48% in the study group and 54% in the control group. Baseline demographics of the study population are outlined in Table 1. All participants were above the legal drinking age and have regularly consumed alcohol prior to this trial, therefore being classified as “social drinkers”. Participants were matched into the different groups as comparable triplets according to baseline characteristics. Therefore, the volunteers’ age, sex, body composition or preference for either beer or wine were similar between the study and control groups.

**Table 1:** Clinical characteristics of the study sample

	**Study group **(*n* = 62)	**Control group **(*n* = 28)
Age, years	24.0 ± 3.5	23.6 ± 3.7
Female sex, %	48.4	53.6
Body weight, kg	70.6 ± 11.0	69.8 ± 10.5
Body fat, %	17.8 ± 6.2	18.0 ± 7.2
Height, cm	177.5 ± 8.4	176.1 ± 8.6
BMI, kg/m^2^	22.3 ± 2.0	22.4 ± 1.9
Alcohol consumption rate*	2.7 ± 0.9	2.8 ± 0.9

Demographic details for the study group (max/min/IQR): age = 40/19/3; body weight = 94/52/15.5; height = 196/163/13.8; BMI = 26.9/17.6/2.8; body fat percentage = 29.3/5/10.4

Demographic details for the control group (max/min/IQR): age = 36/19/4; body weight = 90/53/15.8; height = 193/161/13.5; BMI = 27.8/19.7/2.6; body fat percentage = 29.9/5.3/12.3

*BMI* body-mass-index, *IQR* interquartile range, *SD* standard deviation

*0 = rarely, 1 = once monthly, 2 = more than once monthly and less than once weekly, 3 = once weekly, 4 = more than once weekly; data are presented as means ± SD

**Fig. 2:**
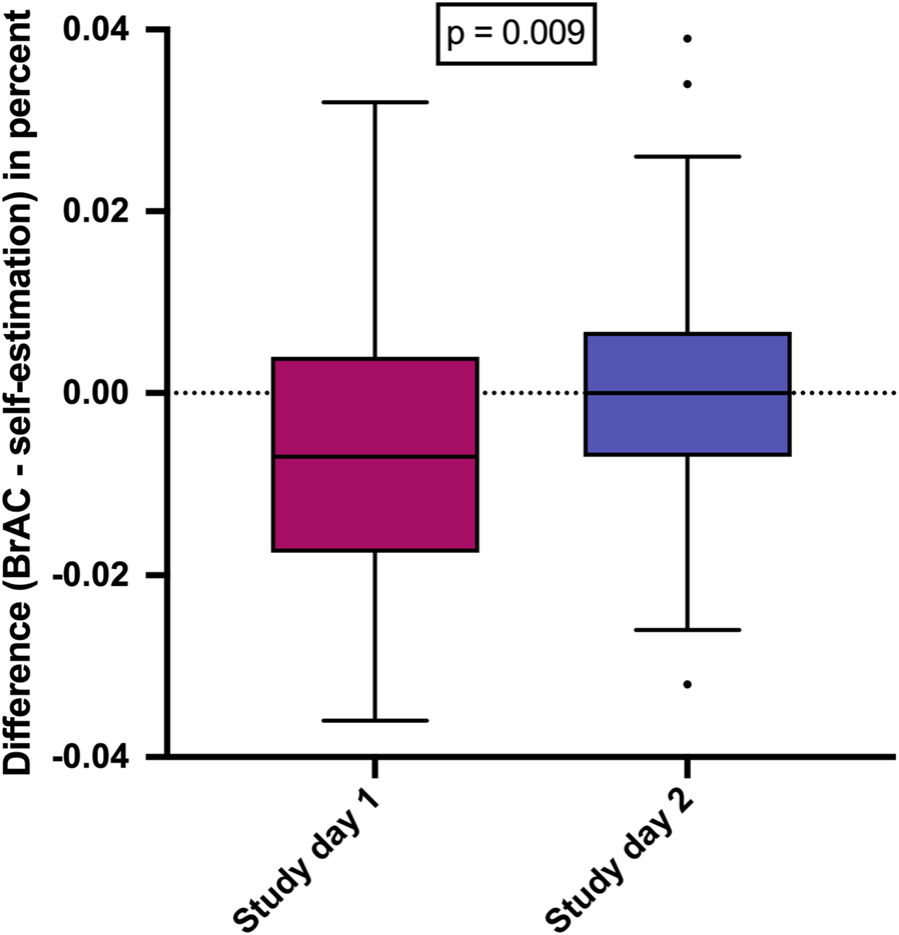
Comparison of self-estimation accuracy between the two intervention days. To evaluate the accuracy of drinkers’ self-estimation, the volunteers were asked to report when they perceived to have reached the legal driving limit of 0.05% BrAC. Data show the difference between measured BrAC and self-estimated BrAC. The p value was calculated using a paired t test. Summary statistics: Study day 1: − 0.036/ − 0.017/ − 0.007/ 0.004/ 0.032 (Min/ Q1/ Median/ Q2/ Max); study day 2 = − 0.032/ − 0.007/ 0.000/ 0.006/ 0.039 (Min/ Q_1_/ Median/ Q_2_/ Max). *BrAC* breath alcohol concentration

Figure 2 illustrates 136 measurements of 68 volunteers who reported to have exactly reached the legal driving limit. In addition, Table 2 displays data of 202 measurements from all 90 volunteers, including all estimations when participants reported to have reached the limit, as well as when they believed to be even below that limit. Our study showed that overall, the participants’ ability to correctly estimate their own level of alcohol intoxication and fitness to operate a motor vehicle was limited. Whilst 61% of all participants who believed they had reached the legal driving limit demonstrated correct or safe self-estimation of their current alcohol intoxication levels on study day 1 (47% on study day 2), a substantial share of volunteers incorrectly estimated their ability to legally operate a motor vehicle (safety concern). On study day 1, 39% of participants who believed to have reached the legal driving limit (perceived BrAC = 0.05%) had in fact already exceeded this threshold (measured BrAC = 0.062 ± 0.01). On study day 2, this proportion increased to 53% (BrAC 0.059 ± 0.007). Nine out of 12 times (75%) a volunteer who believed to still have a BrAC below the legal driving limit had in fact already passed the legal threshold (BrAC = 0.064 ± 0.018) on study day one (42% on study day 2, BrAC = 0.075 ± 0.015). Altogether, on both study days, 96 inaccurate self-estimations were provided by participants believing to have reached the legal driving limit or be still below the limit whilst already exceeding that threshold (Additional file 1: Fig. S1).

**Table 2:** Participants’ self-assessment regarding being legally permitted to drive a car

		Study day 1		Study day 2	
		Correct/safe self-estimation	Incorrect self-estimation (safety concern)	Correct/safe self-estimation	Incorrect self-estimation (safety concern)
“I have reached the legal driving limit” (self-estimated BrAC 0.05%)	*n* (%)	52 (61.2%)	33 (38.8%)	44 (47.3%)	49 (52.7%)
	Measured BrAC (mean ± SD)	0.035 ± 0.009	0.062 ± 0.01	0.04 ± 0.009	0.059 ± 0.007
“I am below the legal driving limit” (self-estimated BrAC < 0.05%)	*n* (%)	3 (25.0%)	9 (75.0%)	7 (58.3%)	5 (41.6%)
	Measured BrAC (mean ± SD)	0.045 ± 0.003	0.064 ± 0.018	0.041 ± 0.008	0.075 ± 0.015

*BrAC* breath alcohol concentration, *SD* standard deviation

To evaluate whether a learning effect can be observed between the two study participation days, we compared the volunteers’ self-estimation of having reached the legal driving limit between the two study days (Fig. 2). On study day two, self-estimation was overall more accurate as compared to study participation day 1 (mean intraindividual difference between measured and estimated BrAC values ± SD = 0.000 ± 0.013 vs. − 0.005 ± 0.015; *p* = 0.009). However, no major differences between self-estimation accuracy of study days 1 and 2 could be detected when examining study and control group separately (Additional file 1: Fig. S2).

The type of consumed alcoholic beverage (beer vs. wine, Additional file 1: Fig. S3) showed no meaningful influence on the participants’ self-estimation accuracy on study day 2. On study day one, wine consumption was associated with a slightly more accurate self-estimation as compared to beer consumption. Women were considerably more likely to misjudge having passed the legal driving limit than men on study day 2 (*p* = 0.009). However, on day 1, no striking difference could be detected between women and men (Additional file 1: Fig. S4).

**Fig. 3:**
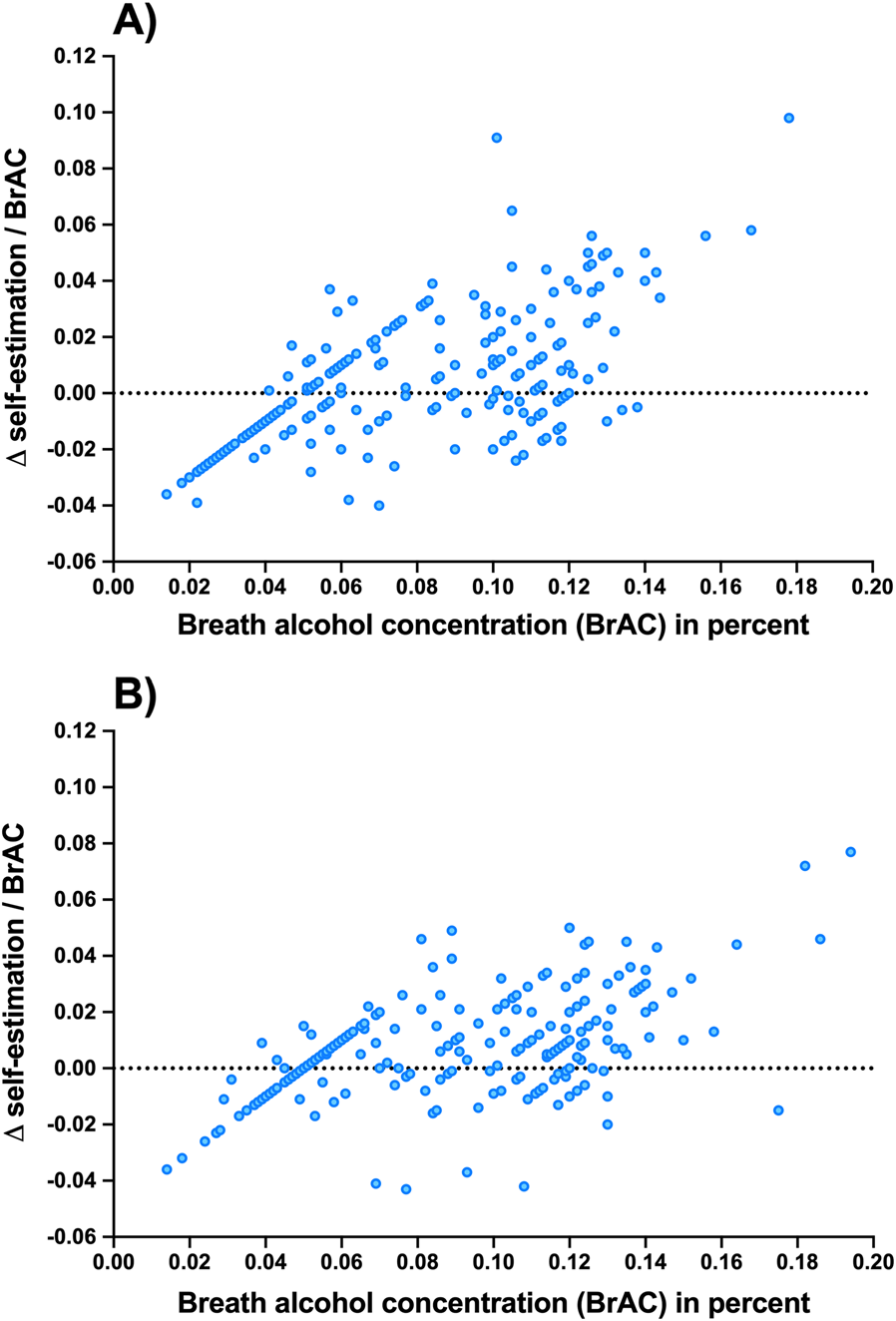
Accuracy of self-estimation in relation to measured breath alcohol concentration (BrAC). Data are presented as differences between measured BrAC and self-estimated BrAC. Negative values represent an overestimation, positive values indicate an underestimation of the objectively detected BrAC. The striking diagonals that have a y-intercept of − 0.05 and a slope of 1 arise from participants estimating their BrAC as 0.05, and all BrAC measurements being recorded to two decimals. Study day 1 is shown in **a**, study day 2 in **b**

Further, we visualised 234 measurements for study day 1 and 248 measurements on study day 2 regarding the accuracy of the volunteers’ self-estimations in relation to their level of alcohol intoxication (Fig. 3). A strikingly positive correlation can be observed with estimations being less accurate with increasing BrAC levels on study day 1 and study day 2. In detail, on both study participation days, there was an overall tendency to underestimate the degree of alcohol intoxication. Importantly, with increasing BrAC levels, participants increasingly underestimated how drunk they were.

**Fig. 4:**
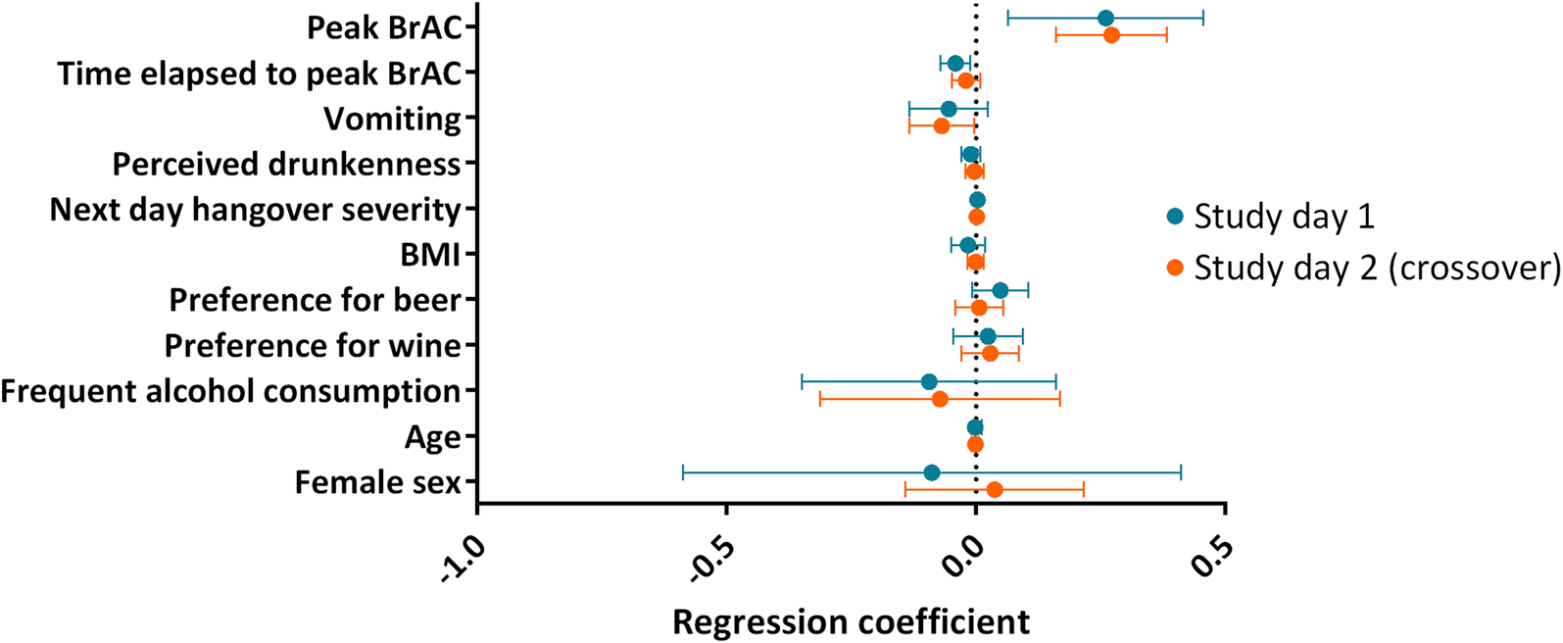
Repeated measurements ANCOVA with fixed and random effects for both study days. The target variable was the subjects’ estimation of current BrAC (absolute difference of measured and perceived BrAC). Estimates for fixed effects are presented including 95% confidence intervals. *BMI* body mass index, *BrAC* breath alcohol concentration.

Next, we sought to identify risk factors for inaccurate self-estimation (safety concern) of acute alcohol intoxication. Type III fixed effect tests identified an impact of peak BrAC, and time elapsed to peak BrAC on the accuracy of self-estimation on study day 1 (peak BrAC and vomiting on study day 2) (Fig. 4; Additional file 1: Tables S1–S4). A higher peak BrAC led to a significantly higher inaccuracy of self-estimation on both study days. Time elapsed to reach the maximum level (study day 1) and vomiting (study day 2) was associated with less inaccurate self-estimation. Other fixed effects like age, sex, body-mass-index, frequency of alcohol consumption or of hangover occurrence, as well as personal alcoholic beverage preferences, were inconspicuous regarding the participants’ self-estimation accuracy.

**Fig. 5:**
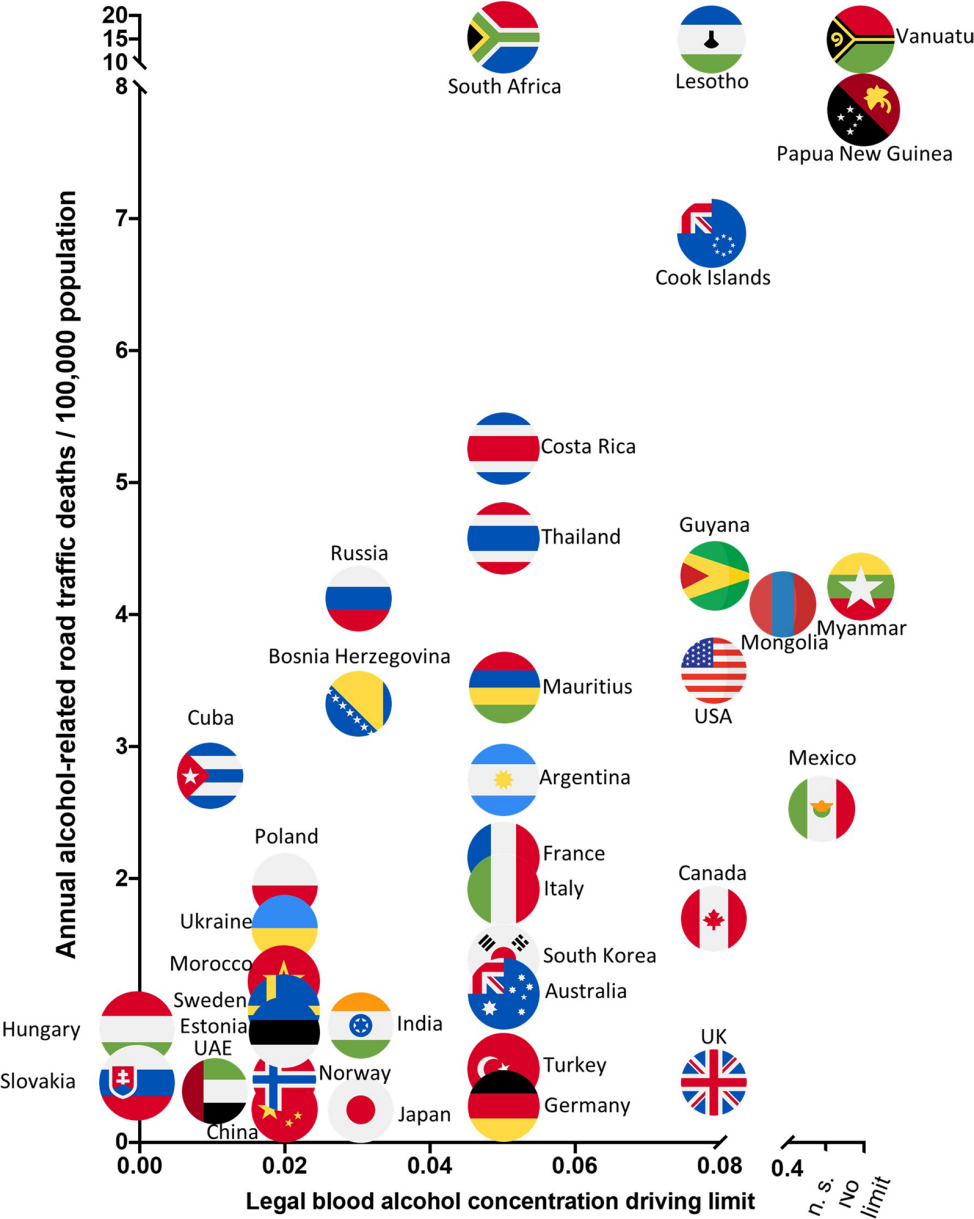
Association of legal blood alcohol concentration driving limits and annual alcohol-related road traffic accident deaths (WHO data). National alcohol-driving limits and alcohol-related road traffic accident deaths were positively correlated (rho = 0.12), a finding that was statistically significant (*p* = 0.03). The *p* value was calculated using linear regression

Finally, to put our findings in a global perspective, we investigated the association of legal blood alcohol concentration driving limits and annual alcohol-related road traffic accident deaths for various countries. We used WHO data [1] for the G20 members, as well as for nations with noticeable legal driving limits or alcohol-related traffic accident deaths (Fig. 5). National driving limits and alcohol-related road traffic accident deaths were positively correlated (rho = 0.12), a finding that was statistically significant (*p* = 0.03).

## Discussion

In this study, we examined social drinkers regarding their ability to correctly estimate the level of intoxication when consuming alcohol. Strikingly, more than one third of all participants believed to be still permitted by law to operate a motor vehicle whilst already exceeding the legal driving limit; and more than half of all even on their second (crossover) study participation (Table 2; Additional file 1: Fig. S1). Acute alcohol intoxication constitutes a significant health risk in numerous ways [15]. Driving under the influence of alcohol is an established major risk factor for road traffic accidents, leading to high levels of morbidity and mortality worldwide [1]. A dose-dependent relationship has been observed for blood alcohol concentrations. Importantly, the risk is already high at low levels of intoxication and estimations indicate the odds of fatal injury to increase by 1.7 for every 0.02% increase in blood alcohol concentration [16]. Moreover, drivers who self-estimate lower blood alcohol concentrations have been shown to exhibit riskier driving behaviours in a placebo-controlled study [17]. This is in line with the previous observation of dangerous attitudes regarding road laws and driving safety that seem to precede traffic road safety violations [18], which renders our observation particularly worrisome.

Interestingly, self-estimations became more accurate on study day 2 (Fig. 2). This indicates that a learning effect may be used to improve self-estimation of social drinkers by making them aware of the issue. This is in line with several studies which demonstrated that self-estimating the degree of alcohol intoxication can in fact be learned [19, 20]. Importantly, our study was not particularly designed to train participants. Further, study group subjects who received information about having reached the legal driving limit did not do better than control group subjects who were blinded about passing the legal limit threshold. Therefore, it is likely the sheer awareness of the issue that improved self-estimation accuracy from study day 1 to study day 2 in this experiment. This could be combined with more specific training techniques [20], particularly targeted for “under-estimators” [21], to enable social drinkers to make more informed decisions when considering participating in road traffic after consuming alcohol. Martí-Belda et al. have compared sociodemographic, personality and alcohol consumption profiles in three groups of vehicle drivers [22]. The analysis revealed distinct personality profiles that differed between offenders banned from driving by penalty points and offenders that were specifically banned from driving by a court order. The repeated violation of a myriad of road traffic laws (such as risky driving) was associated with the unfortunate ‘*immunity*’ regarding re-education and awareness-rising. More research is needed to improve the efficacy of self-estimation learning techniques and, importantly, to identify those who are likely to benefit the most from these interventions.

In the present study, women were more likely to misjudge their alcohol intoxication regarding the legal driving limit on study day 2 (*p* = 0.009). In contrast, on study day 1, this was not statistically significant. The latter is in keeping with other large investigations, which revealed no sex-specific differences regarding self-estimation of BrAC [23]. We would therefore interpret the here observed slight difference with caution and refrain from generalization. Similarly, the type of consumed alcohol, beer vs. wine, did not show a significant effect on the participants self-estimation behaviour. This is in line with a previous study showing the type of alcoholic drink had no influence on the participants’ ability to correctly self-estimate their alcohol intoxication level [24].

In this study, we demonstrated that the tendency to dangerously underestimate the own degree of alcohol intoxication rises with increasing alcohol consumption (Fig. 3). To our knowledge, this is the first study to prove this phenomenon under experimental study conditions with an internal validation using a crossover trial design. These results are in keeping with previous observational studies in naturalistic drinking settings, in which social/college drinkers were approached at night in town or whilst returning from alcohol-serving establishments [11, 13, 25]. Therefore, the enhanced misperception of one’s intoxication level with increasing alcohol consumption is particularly worrisome for countries that permit motor vehicle road traffic participation at higher BrAC levels. To put our findings in context globally, we enquired and visualised WHO data [1] for alcohol-related road traffic deaths and national legal driving limits for numerous countries (Additional file 1: Fig. S5). Applying the German legal limit of 0.05% in this study, we found more than one third of the study participants to misperceive their ability to legally drive a car after limited alcohol consumption. Based on our findings of increasing misjudgement with rising breath alcohol levels, nations with higher legal driving limits such as the US, the UK (except Scotland) or Canada (0.08% for all) are likely to see much higher proportions of social drinkers who misinterpret their degree of intoxication and, thus, their ability to legally participate in road traffic. This given, it is not surprising that nations with no legal driving limit at all (Myanmar, Papua New Guinea or Vanuatu) are among those facing the highest numbers in alcohol-related road traffic deaths worldwide [1].

A limitation of this study was that its experimental, semi-naturalistic setting. Participants were under constant surveillance and underwent serial BrAC measurements and self-estimations. Therefore, our results may not be identical in a naturalistic drinking environment. On the other hand, a strength of this study was the availability of serial measurements (vs. most naturalistic studies that often only deliver isolated, single measurements) and the internal validation using a crossover study design.

## Conclusions

In summary, when consuming alcohol, social drinkers are frequently unaware of exceeding the legal driving limit. However, awareness can improve self-estimating the degree of alcohol intoxication. This is an opportunity to address the avoidable hazard of alcohol-related road traffic accidents. For instance, social media campaigns could actively inform social drinkers about the potential of self-awareness training and the implied personal advantages such as improved safety. Further, dedicated awareness programs could be advertised in pubs, bars or night clubs, and government advice communications could reach out to secondary schools and universities. Specifically, this could be accompanied by mobile ‘pop-up self-awareness training centres’, where pedestrian social drinkers could be educated and, if desired, instantly undergo self-awareness training on a usual night out in a real-world scenario. Finally, gamification features (e.g. in form of a smartphone application add-on) could be implied to improve adherence and popularity of the self-awareness training intervention.

## CONSORT statement

This manuscript and the underlying clinical trial were designed in accordance with the CONSORT statement for evidence-based, complete and transparent reporting of randomised trial. The corresponding CONSORT checklist can be accessed in the Additional file 2.

## Supplementary Information

**Additional file 1.** Supplementary data.

**Additional file 2.** CONSORT checklist.

## Data Availability

The datasets generated during and/or analysed during the current study are available from the corresponding author on reasonable request.
