# Supplementary material for: The hazardous (mis)perception of Self-estimated Alcohol intoxication and Fitness to drivE—an avoidable health risk: the SAFE randomised trial
Source: Harm Reduct J. 2021 Dec 7;18:122. doi: 10.1186/s12954-021-00567-4 (PMC8650558; doi:10.1186/s12954-021-00567-4)
Supplement: Supplementary file 1 — Additional file 1. Supplementary data. [file 12954_2021_567_MOESM1_ESM.docx]

**Additional figures and tables**


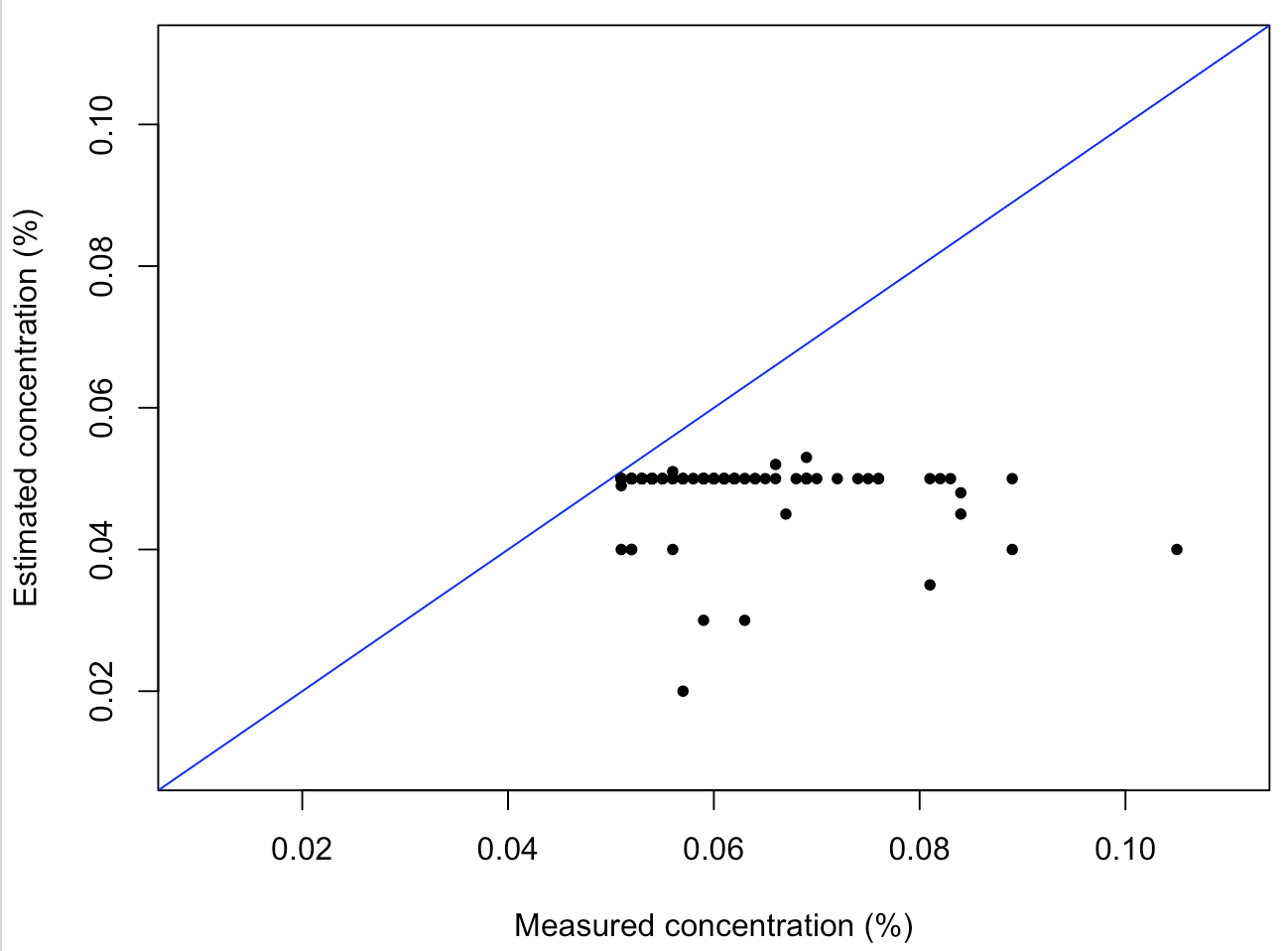


**Additional Figure S1.** Scatterplot demonstrating breath alcohol concentration self-estimations and measurements. In total, 96 estimations yielded “concerning” misjudgements on both study days. Each dot represents a participant who believed to have reached the legal driving limit or be still below the driving limit while already having exceeded this threshold.

**
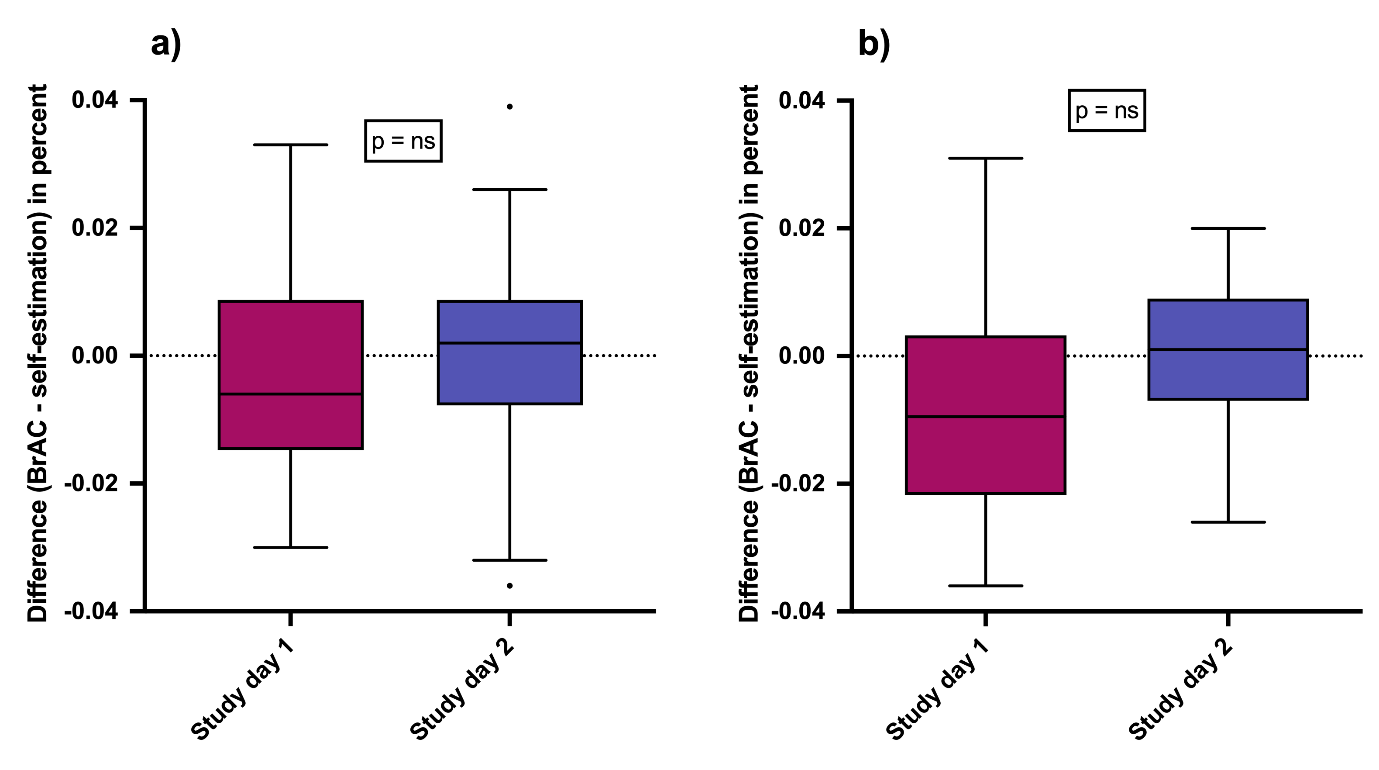
**

**Additional figure S2.** Comparison of self-estimation accuracy between the two intervention days for the study group (**a**) and the control group (**b**). To evaluate the accuracy of drinkers’ self-estimation, the volunteers were asked to report when they perceived to have reached the legal driving limit of 0.05% BrAC. Data shows the difference between measured BrAC and self-estimated BrAC. P-values were calculated using unpaired t-tests.
BrAC breath alcohol concentration.


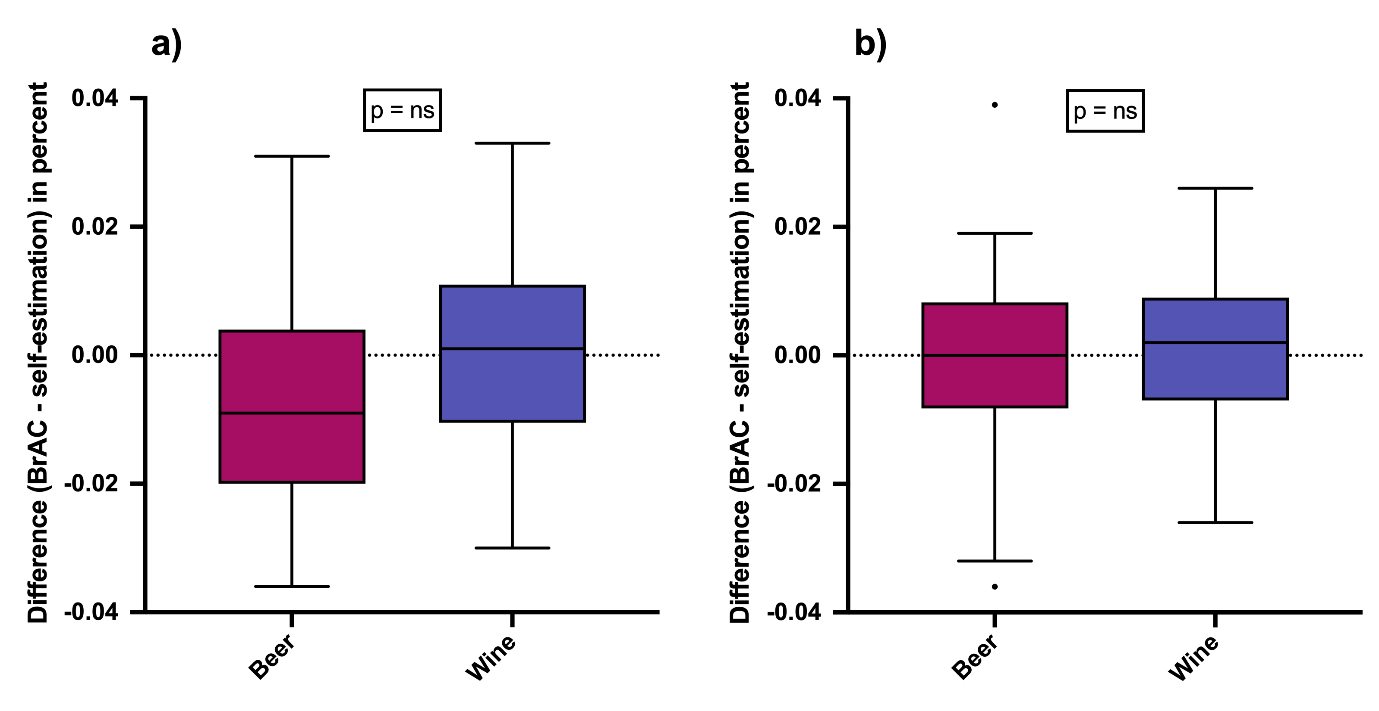


**Additional figure S3.** Comparison of self-estimation accuracy in relation to the consumed alcoholic beverages for study day one (**a**) and study day two (**b**). To evaluate the accuracy of drinkers’ self-estimation, the volunteers were asked to report when they perceived to have reached the legal driving limit of 0.05% BrAC. Data shows the difference between measured BrAC and self-estimated BrAC. P-values were calculated using unpaired t-tests.
BrAC = breath alcohol concentration.


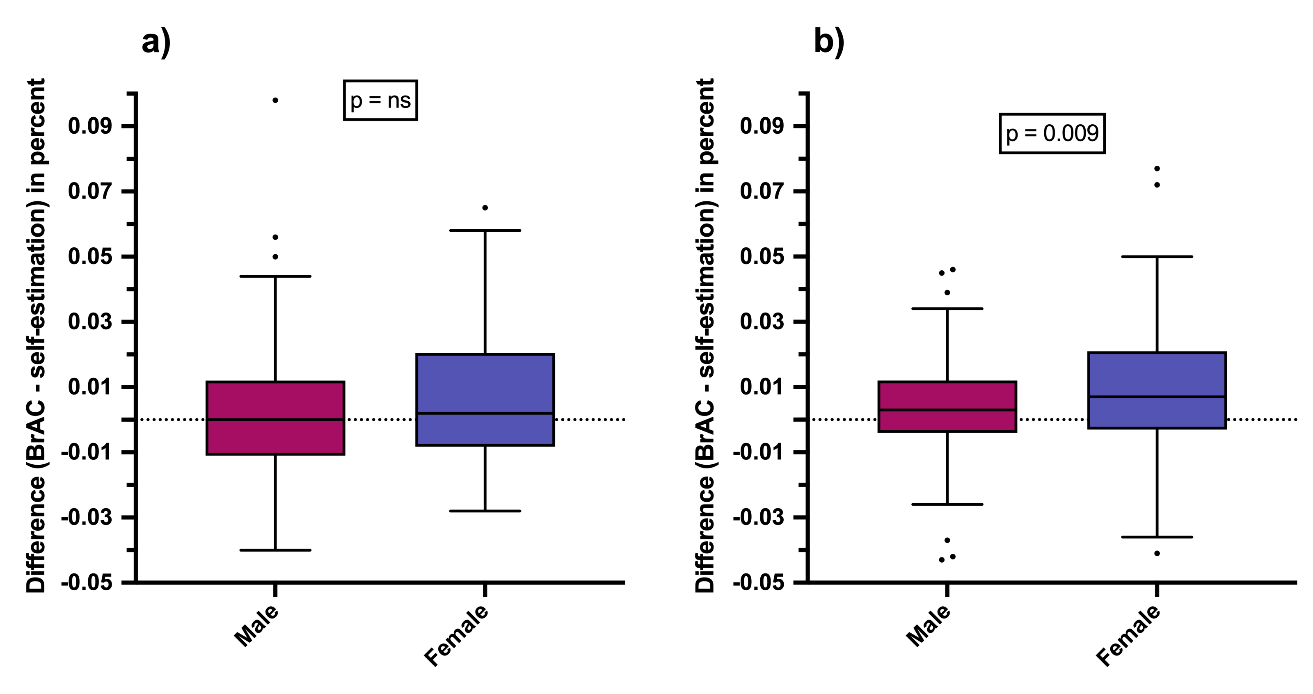


**Additional figure S4.** Comparison of self-estimation accuracy in relation to sex for study day one (**a**) and study day two (**b**). To evaluate the accuracy of drinkers’ self-estimation, the volunteers were asked to report when they perceived to have reached the legal driving limit of 0.05% BrAC. Data shows the difference between measured BrAC and self-estimated BrAC. P-values were calculated using unpaired t-tests.
BrAC = breath alcohol concentration.

**Additional table S1.** This table shows Type III tests of fixed effects including numerator and denominator degrees of freedom (DF), F-values and p-values for study day one. The analysis was performed with SAS version 9.4 using PROC GLIMMIX.

| **Type III tests of fixed effects** | | | | |
| --- | --- | --- | --- | --- |
| **Effect** | **Num DF** | **Den DF** | **F-value** | **p-value** |
| **Time point** | 4 | 169.5 | 6.39 | <.0001 |
| **Order of beverage consumption** | 3 | 61.05 | 0.69 | 0.5611 |
| **Sex** | 1 | 66.556 | 0.13 | 0.7240 |
| **Age** | 1 | 62.38 | 0.07 | 0.7983 |
| **Preferred beverage** | 2 | 65.87 | 1.53 | 0.2248 |
| **Alcohol consumption frequency** | 4 | 59.7 | 0.86 | 0.4962 |
| **Hangover frequency** | 3 | 61.33 | 0.32 | 0.8103 |
| **Medications** | 1 | 68 | 2.13 | 0.1493 |
| **Amount of food consumed** | 1 | 62.18 | 0.23 | 0.6302 |
| **Time elapsed to peak BrAC** | 1 | 58.95 | 7.65 | 0.0076 |
| **Peak BrAC** | 1 | 60.54 | 7.04 | 0.0102 |
| **Vomiting** | 1 | 50.33 | 1.95 | 0.1687 |
| **Percent body fat** | 1 | 61.31 | 0.30 | 0.5846 |
| **Percent body water** | 1 | 59.82 | 0.26 | 0.6094 |
| **Percent body muscle** | 1 | 70.34 | 0.11 | 0.7406 |
| **BMI** | 1 | 65.61 | 0.85 | 0.3598 |
| **Alcohol hangover scale** | 1 | 62.1 | 2.48 | 0.1203 |
| **Perceived drunkenness** | 1 | 52.59 | 1.22 | 0.2737 |

**Additional table S2.** This table shows Type III tests of fixed effects including numerator and denominator degrees of freedom (DF), F-values and p-values for study day two. The analysis was performed with SAS version 9.4 using PROC GLIMMIX.

| **Type III tests of fixed effects** | | | | |
| --- | --- | --- | --- | --- |
| **Effect** | **Num DF** | **Den DF** | **F-value** | **p-value** |
| **Time point** | 4 | 173.4 | 8.39 | <.0001 |
| **Order of beverage consumption** | 3 | 49.65 | 0.64 | 0.5919 |
| **Sex** | 1 | 46.06 | 0.18 | 0.6725 |
| **Age** | 1 | 43.51 | 0.06 | 0.8069 |
| **Preferred beverage** | 2 | 53.89 | 0.50 | 0.6111 |
| **Alcohol consumption frequency** | 4 | 46.8 | 0.57 | 0.6831 |
| **Hangover frequency** | 3 | 54.73 | 0.67 | 0.5733 |
| **Medications** | 1 | 52.12 | 0.00 | 0.9925 |
| **Amount of food consumed** | 1 | 56.85 | 0.45 | 0.5046 |
| **Time elapsed to peak BrAC** | 1 | 53.43 | 2.02 | 0.1612 |
| **Peak BrAC** | 1 | 49.01 | 24.38 | <.0001 |
| **Vomiting** | 1 | 47.01 | 4.40 | 0.0414 |
| **Percent body fat** | 1 | 52.36 | 0.33 | 0.5700 |
| **Percent body water** | 1 | 52.54 | 0.43 | 0.5157 |
| **Percent body muscle** | 1 | 44.62 | 0.70 | 0.4057 |
| **BMI** | 1 | 50.77 | 0.00 | 0.9643 |
| **Alcohol hangover scale** | 1 | 46.63 | 0.84 | 0.3639 |
| **Perceived drunkenness** | 1 | 58.36 | 0.11 | 0.7404 |

**Additional table S3.** Repeated measurements ANCOVA with fixed and random effects for study day one: Comprehensive analysis showing solutions for fixed effects including estimated regression coefficients with standard errors, 95% confidence intervals as well as degrees of freedom, t-values and p-values of Wald test (alpha = 0.05).

| **Effect** | **Category** | **Reference** | **Estimate** | **Standard error** | **DF** | **t-value** | **p-value** | **Alpha** | **Lower CI** | **Upper CI** |
| --- | --- | --- | --- | --- | --- | --- | --- | --- | --- | --- |
| **Intercept** |  |  | -1.8595 | 4.5526 | 62.91 | -0.41 | 0.6843 | 0.05 | -10.9573 | 7.2384 |
| **Time point** | 2 | 1 | 0.06953 | 0.02376 | 162.5 | 2.93 | 0.0039 | 0.05 | 0.02260 | 0.1165 |
| **Time point** | 3 | 1 | 0.1213 | 0.02535 | 166.7 | 4.79 | <.0001 | 0.05 | 0.07125 | 0.1713 |
| **Time point** | 4 | 1 | 0.09259 | 0.0616 | 180.3 | 2.56 | 0.0113 | 0.05 | 0.02124 | 0.1639 |
| **Time point** | 5 | 1 | 0.09196 | 0.07744 | 172.3 | 1.19 | 0.02367 | 0.05 | -0.06090 | 0.2448 |
| **Order of beverage consumption** | Beer-wine | Beer | -0.04661 | 0.03249 | 61.72 | -1.43 | 0.1565 | 0.05 | -0.1116 | 0.01835 |
| **Order of beverage consumption** | Wine | Beer | -0.02908 | 0.05025 | 57.44 | -0.58 | 0.5650 | 0.05 | -0.1297 | 0.04730 |
| **Order of beverage consumption** | Wine-beer | Beer | -0.02208 | 0.03476 | 66.77 | -0.64 | 0.5274 | 0.05 | -0.09146 | 0.04730 |
| **Sex** | Female | Male | -0.08868 | 0.2501 | 66.55 | -0.35 | 0.7240 | 0.05 | 0.5880 | 0.4106 |
| **Age** |  |  | -0.00170 | 0.006624 | 62.38 | -0.26 | 0.7983 | 0.05 | -0.01494 | 0.01154 |
| **Preferred beverage** | Beer | No preference | 0.04910 | 0.02811 | 76.49 | 1.75 | 0.0847 | 0.05 | -0.00688 | 0.1051 |
| **Preferred beverage** | Wine | No preference | 0.02396 | 0.03488 | 57.94 | 0.69 | 0.4949 | 0.05 | -0.04587 | 0.09378 |
| **Alcohol consumption frequency** | 1/month | Rarely | -0.08467 | 0.1279 | 55.9 | -0.66 | 0.5107 | 0.05 | -0.3409 | 0.1715 |
| **Alcohol consumption frequency** | >1/month | Rarely | -0.03817 | 0.1237 | 56.13 | -0.31 | 0.7588 | 0.05 | -0.2859 | 0.2096 |
| **Alcohol consumption frequency** | 1/week | Rarely | -0.08762 | 0.1245 | 60.56 | -0.70 | 0.4841 | 0.05 | -0.3365 | 0.1613 |
| **Alcohol consumption frequency** | >1/week | Rarely | -0.09415 | 0.1272 | 58.22 | -0.74 | 0.4620 | 0.05 | -0.3487 | 0.1604 |
| **Hangover frequency** | 1/month | Rarely | -0.00166 | 0.03537 | 67.84 | -0.05 | 0.9627 | 0.05 | -0.07224 | 0.06892 |
| **Hangover frequency** | >1/month | Rarely | -0-00563 | 0.03935 | 65.64 | -0.14 | 0.8867 | 0.05 | -0.08420 | 0.07295 |
| **Hangover frequency** | 1/week | Rarely | 0.03148 | 0.04846 | 65.33 | 0.65 | 0.5182 | 0.05 | -0.06529 | 0.1283 |
| **Medications** | Yes | No | -0.05010 | 0.03435 | 68 | -1.46 | 0.1493 | 0.05 | -0.1186 | 0.01845 |
| **Amount of food consumed** |  |  | 0.000074 | 0.000153 | 62.18 | 0.48 | 0.6302 | 0.05 | -0.00023 | 0.000380 |
| **Time elapsed to max BrAC** |  |  | -0.04131 | 0.01493 | 58.95 | -2.77 | 0.0076 | 0.05 | -0.07119 | -0.01143 |
| **Max BrAC** |  |  | 0.2602 | 0.09807 | 60.54 | 2.65 | 0.0102 | 0.05 | 0.06402 | 0.4563 |
| **Vomiting** | Yes | No | -0.05457 | 0.03908 | 50.33 | -1.40 | 0.1687 | 0.05 | -0.1331 | 0.02391 |
| **Percent body fat** |  |  | 0.02879 | 0.05240 | 61.31 | 0.55 | 0.5846 | 0.05 | -0.07597 | 0.1336 |
| **Percent body water** |  |  | 0.03589 | 0.06989 | 59.82 | 0.51 | 0.6094 | 0.05 | -0.1039 | 0.1757 |
| **Percent body muscle** |  |  | -0.00934 | 0.02812 | 70.34 | -0.33 | 0.7406 | 0.05 | -0.06542 | 0.04673 |
| **BMI** |  |  | -0.01565 | 0.01697 | 65.61 | -0.92 | 0.3598 | 0.05 | -0.04954 | 0.01824 |
| **Alcohol hangover scale** |  |  | 0.002793 | 0.001773 | 62.1 | 1.58 | 0.1203 | 0.05 | -0.00075 | 0.006338 |
| **Perceived drunkenness** |  |  | -0.01053 | 0.009523 | 52.59 | -1.11 | 0.2737 | 0.05 | -0.02964 | 0.008569 |

**Additional table S4.** Repeated measurements ANCOVA with fixed and random effects for study day two: Comprehensive analysis showing solutions for fixed effects including estimated regression coefficients with standard errors, 95% confidence intervals as well as degrees of freedom, t-values and p-values of Wald test (alpha = 0.05).

| **Effect** | **Category** | **Reference** | **Estimate** | **Standard error** | **DF** | **t-value** | **p-value** | **Alpha** | **Lower CI** | **Upper CI** |
| --- | --- | --- | --- | --- | --- | --- | --- | --- | --- | --- |
| **Intercept** |  |  | 2.9662 | 5.4322 | 52.43 | 0.55 | 0.5874 | 0.05 | -7.9322 | 13.8646 |
| **Time point** | 2 | 1 | 0.07770 | 0.01761 | 160 | 4.41 | <.0001 | 0.05 | 0.04293 | 0.1125 |
| **Time point** | 3 | 1 | 0.1033 | 0.01993 | 172.9 | 5.18 | <.0001 | 0.05 | 0.06398 | 0.1427 |
| **Time point** | 4 | 1 | 0.04875 | 0.02963 | 183.3 | 1.65 | 0.1016 | 0.05 | -0.00971 | 0.1072 |
| **Time point** | 5 | 1 | 0.09584 | 0.05132 | 179.9 | 1.87 | 0.0635 | 0.05 | -0.00543 | 0.1971 |
| **Order of beverage consumption** | Beer-wine | Beer | 0.03713 | 0.04161 | 45.19 | 0.89 | 0.3770 | 0.05 | -0.04667 | 0.1209 |
| **Order of beverage consumption** | Wine | Beer | 0.02339 | 0.04555 | 48.03 | 0.51 | 0.6100 | 0.05 | -0.06820 | 0.1150 |
| **Order of beverage consumption** | Wine-beer | Beer | -0.00175 | 0.03762 | 42.73 | -0.05 | 0.9632 | 0.05 | -0.07764 | 0.07414 |
| **Sex** | Female | Male | 0.03778 | 0.08882 | 46.06 | 0.43 | 0.6725 | 0.05 | -0.1410 | 0.2166 |
| **Age** |  |  | -0.00092 | 0.003745 | 43.51 | -0.25 | 0.8069 | 0.05 | -0.00847 | 0.006629 |
| **Preferred beverage** | Beer | No preference | 0.006661 | 0.02379 | 55.58 | 0.28 | 0.7805 | 0.05 | -0.04100 | 0.05432 |
| **Preferred beverage** | Wine | No preference | 0.02874 | 0.02891 | 53.75 | 0.99 | 0.3246 | 0.05 | -0.02922 | 0.08671 |
| **Alcohol consumption frequency** | 1/month | Rarely | -0.01707 | 0.1152 | 44.89 | -0.15 | 0.8829 | 0.05 | -0.2491 | 0.2149 |
| **Alcohol consumption frequency** | >1/month | Rarely | -0.03376 | 0.1131 | 45.33 | -0.30 | 0.7667 | 0.05 | -0.2615 | 0.1940 |
| **Alcohol consumption frequency** | 1/week | Rarely | -0.03203 | 0.1174 | 45.94 | -0.27 | 0.7862 | 0.05 | -0.2684 | 0.2043 |
| **Alcohol consumption frequency** | >1/week | Rarely | -0.07232 | 0.1196 | 47.87 | -0.60 | 0.5484 | 0.05 | -0.3129 | 0.1682 |
| **Hangover frequency** | 1/month | Rarely | 0.03877 | 0.03097 | 56.41 | 1.25 | 0.2158 | 0.05 | -0.02327 | 0.1008 |
| **Hangover frequency** | >1/month | Rarely | 0.03261 | 0.03201 | 51.47 | 1.02 | 0.3131 | 0.05 | -0.03164 | 0.09685 |
| **Hangover frequency** | 1/week | Rarely | 0.01470 | 0.04315 | 54.45 | 0.34 | 0.7347 | 0.05 | -0.07180 | 0.1012 |
| **Medications** | Yes | No | -0.00024 | 0.02542 | 52.12 | -0.01 | 0.9925 | 0.05 | -0.05125 | 0.05077 |
| **Amount of food consumed** |  |  | -0.00007 | 0.000112 | 56.85 | -0.67 | 0.5046 | 0.05 | -0.00030 | 0.000149 |
| **Time elapsed to max BrAC** |  |  | -0.02022 | 0.01423 | 53.43 | -1.42 | 0.1612 | 0.05 | -0.04876 | 0.008322 |
| **Max BrAC** |  |  | 0.2718 | 0.05505 | 49.01 | 4.94 | <.0001 | 0.05 | 0.1612 | 0.3824 |
| **Vomiting** | Yes | No | -0.06845 | 0.03264 | 47.01 | -2.10 | 0.0414 | 0.05 | -0.1341 | -0.00279 |
| **Percent body fat** |  |  | -0.03185 | 0.05571 | 52.36 | -0.57 | 0.5700 | 0.05 | -0.1436 | 0.07992 |
| **Percent body water** |  |  | -0.04804 | 0.07340 | 52.54 | -0.65 | 0.5157 | 0.05 | -0.1953 | 0.09921 |
| **Percent body muscle** |  |  | 0.008165 | 0.009727 | 44.62 | 0.84 | 0.4057 | 0.05 | -0.01143 | 0.02776 |
| **BMI** |  |  | -0.00036 | 0.008019 | 50.77 | -0.04 | 0.9643 | 0.05 | -0.01646 | 0.01574 |
| **Alcohol hangover scale** |  |  | 0.001368 | 0.001492 | 46.63 | 0.92 | 0.3639 | 0.05 | -0.00163 | 0.004371 |
| **Perceived drunkenness** |  |  | -0.00305 | 0.009168 | 58.36 | -0.33 | 0.7404 | 0.05 | -0.02140 | 0.01530 |
